# Adaptation of an amphibian mucociliary clearance model to evaluate early effects of tobacco smoke exposure

**DOI:** 10.1186/1465-9921-5-9

**Published:** 2004-08-20

**Authors:** J Gustavo Zayas, Darryl W O'Brien, Shusheng Tai, Jie Ding, Leonard Lim, Malcolm King

**Affiliations:** 1 Mucobiology Research Unit, Pulmonary Research Group, University of Alberta, Edmonton, Canada

## Abstract

**Rationale:**

Inhaled side-stream tobacco smoke brings in all of its harmful components impairing mechanisms that protect the airways and lungs. Chronic respiratory health consequences are a complex multi-step silent process. By the time clinical manifestations require medical attention, several structural and functional changes have already occurred. The respiratory system has to undergo an iterative process of injury, healing and remodeling with every exposure.

**Methods:**

To have a better understanding of the initial changes that take place when first exposed to environmental tobacco smoke, we have developed an exposure model, using the frog palate that closely represents the features of obstructive airways where ciliary dysfunction and mucus hypersecretion occur.

**Results:**

Mucus transport was significantly reduced, even after exposure to the smoke of one cigarette (p < 0.05) and even further with 4-cigarettes exposure (p < 0.001). Morphometric and ultrastructural studies by SEM show extensive areas of tissue disruption. Gelatinase zymography shows activation of MMP9 in mucus from palates exposed to tobacco smoke.

**Conclusions:**

The clearance of mucus on the frog palate is significantly reduced after exposure to environmental tobacco smoke. Cilia and the extracellular matrix are anatomically disrupted. Tobacco smoke triggers an increased activity of matrix metalloproteinases associated with a substantial defoliation of ciliated epithelium. These studies enhance the knowledge of the changes in the mucociliary apparatus that occur initially after exposure to environmental tobacco smoke, with the goal of understanding how these changes relate to the genesis of chronic airway pathologies in humans.

## Background

Respiratory diseases, infectious and non-infectious, are a prime cause of morbidity, mortality and health system utilization in many countries. Exposure to cigarette smoke is an important factor in causing as well as increasing complications in several pulmonary disorders. The mucociliary clearance constitutes the first line of defense to maintain the airways as free as possible of foreign bodies [1]. Impairment of mucociliary function may be the result of epithelial airway damage, ciliary dysfunction, inflammation, and change in mucus viscosity and/or elasticity.

In laboratory studies we have shown that the physical properties of mucus in nonsmokers are not altered by age or by restrictive pulmonary pathology. However, mucus properties show alterations when exposed to tobacco smoke and these alterations are noticeable in the very early stages of smoke exposure, even at an exposure level in the range of 1 to 5 cigarettes per day [2].

The majority of studies on the outcome of tobacco use have been performed in the late phases of the process [3-7]; the acute and early effects on cilia, mucus and mucociliary clearance after active smoking or side stream tobacco smoke exposure have not been well studied. We hypothesized that studying changes occurring in the initial stages will lead to a better understanding of the multifaceted problems of tobacco exposure.

Therefore our laboratory has been developing and using *in vivo *and *ex vivo *epithelial injury models that replicate the features of airway diseases [8-12]. The mucus blanket in the frog palate is cleared by coordinated ciliary activity in an almost identical fashion to that observed in human airways. We anticipated that a modified frog palate exposure model could better serve our purposes. We were specifically interested in developing an exposure model that would allow us to study the initial effects and mechanisms occurring in an epithelial tissue after exposure to environmental tobacco smoke. We conceived this model to be free of interferences from other agents or systemic physiological responses resulting from other internal or external influences.

We also aim to have a better understanding of the mechanism by which ciliated epithelial cells are exfoliated after being exposed to tobacco smoke, as this may relate directly to impaired mucus clearance in several human airway diseases including chronic bronchitis and chronic obstructive pulmonary disease (COPD). Hence we decided first to test our novel *ex vivo *model, and in later studies use *in vivo *models.

## Methodology

### Frog palate preparation

From a bullfrog, *Rana catesbiana*, the upper portion of the head is removed following the procedures described in previous work [10,11], by cutting with scissors through from the junction of the posterior pharynx and esophagus out to the skin of the back. This procedure was carried out after lowering the body temperature of the frog for 30 – 60 minutes inside a refrigerator to abolish pain sensations. The palate was examined for macroscopic lesions, such as ulcers, petechia or redness as evidence of inflammation. Only palates free of inflammatory indicators were included in this study. Any blood remaining in the epithelial surface was carefully washed away, then the excised head was placed palate side facing upwards on a piece of gauze saturated with frog Ringer solution (FRS) in a Petri dish. The experimental procedures involving animals were approved by the Health Sciences Animal Policy and Welfare Committee, University of Alberta.

The FRS was prepared by mixing standard Ringer injection with sterile water (2:1). The composition of standard frog Ringer (in mmol/l) is 90 NaCl, 3 KCl, 2 CaCl_2_, and 15 NaHCO_3_ (220 mosm/l). The palate was placed inside the frog chamber, a wooden box with a glass top and fitted glass front, and manipulated trough glove openings. The humidity inside the box is maintained at 100% using an ultrasonic Pari nebulizer and the temperature is kept between 22° to 24°C by a rheostat-controlled, externally mounted light source. Before carrying out any measurement, the palate was allowed to stabilize inside the box for 15 minutes before testing.

### Exposure chamber

**Figure 1:**
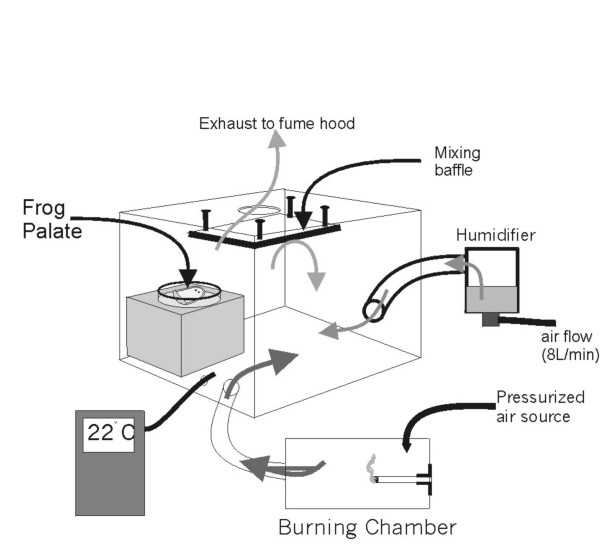
Box designed to expose the frog palate to cigarette smoke.

The exposure chamber (Figure 1) with a volume ~10 liters had two inlets: one connected to an ultrasonic Pari jet nebulizer system set at 8 L/min measured by a Puritan flow meter to maintain the chamber near 100 % humidity. The other inlet was linked to a burning chamber. The latter, which contained a burning cigarette, was slightly pressurized with air flowing into the chamber at a rate of 2 L/min to promote cigarette combustion. Positive ventilation inside the burning chamber pushed the side stream smoke into the covered but not sealed exposure chamber, which was exhausted into a fume hood. Temperature inside the chamber was maintained to 22°C. and monitored through a thermocouple and digital-display thermometer. The palate was placed inside with the palate side upwards on a piece of gauze saturated with FRS in a glass dish at about five centimeters above the bottom of the chamber.

The exposure chamber was designed to allow a steady flow of side stream tobacco smoke to reach the epithelial tissue. The concentration of cigarette smoke inside the exposure chamber was maintained constant throughout the experiment by the presence of a mixing baffle on the outflow of the chamber. The concentration of tobacco smoke inside the chamber was not directly measured, but is estimated that the half palate was exposed to the smoke of each cigarette delivered, diluted in 180 liters of fresh air. As much as possible, the conditions (humidity, type of solution, temperature, physical manipulation and airflow in and out) in the frog chamber and in the exposure chamber were maintained similar, with cigarette smoke being the independent variable.

### Frog palate exposure model preparation

**Figure 2:**
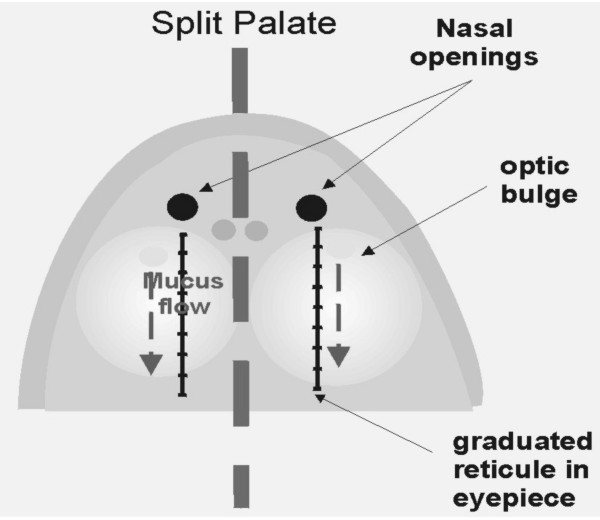
The frog palate was further divided longitudinally in two halves following the midline.

The palate was divided longitudinally in two halves along the midline as shown in Figure 2, cutting the epithelium with a scalpel to minimize damage. After five minutes the mucus transport was measured in both halves, left and right, to confirm that both half palates were functioning normally. One side was used as control and the other half was exposed to tobacco smoke. The control half of the palate was left in the frog chamber while the other half was placed in the exposure chamber maintained at similar conditions of 100 % humidity and room temperature. If any or both halves showed a variation in mucus transport greater than 30 % from the baseline, the experiment was aborted.

### Mucus transport velocity (MTV) determination

The palate was placed under a dissecting stereomicroscope provided with a reticulated eyepiece. Mucociliary clearance was determined by observing the movement of particles of charcoal powder gently deposited on a sample of mucus on the palate surface; its clearance was visually monitored and MTV determined. The displacement of 3 – 5 μL of endogenous frog mucus sample was calculated by dividing the distance traveled by the transit time across the 0.3 inch (7.62 mm) segment marked between 0.1 and 0.4 inches in the graduated eyepiece. At least five measurements of the time required for the mucus sample to travel the defined distance were made every time to obtain control and smoke exposure mucus transport velocity.

We used cigarettes regularly available at commercial outlets of a representative brand in terms of customer preference and toxic emissions: nicotine 0.5 – 2.1 mg, tar 4 – 24 mg, carbon monoxide 5 – 25 mg, hydrogen cyanide 0.04 – 0.21 mg, benzene 0.025 – 0.069 mg, formaldehyde 0.018 – 0.1 mg. One cigarette took 17 minutes on average to burn completely in this preparation.

### Measurements and mucus samples collection

After being exposed to one cigarette, the half palate was brought back to the frog chamber and MTV was measured in both control and exposed halves. A sample of mucus was collected from each half, and placed in separate containers and frozen at -80°C in liquid nitrogen followed by storage in a -80° freezer until analysis. The exposed half, brought back to the exposure chamber was further exposed to 3 more cigarettes (51 additional minutes of exposure). Following tobacco exposure, the half palate was again brought back to the frog chamber to measure mucus transport and collect tissue and mucus sample in both halves, control and exposed. One set of half palates (control and exposed to four cigarettes) was stored at 4°C overnight, and transport of mucus was reassessed the next day.

### Tissue and mucus samples collection

Sample of mucus from both halves were immersed in glutaraldehyde 2.5%, and placed in properly identified separate containers for storage at 4°C for later Scanning Electron Microscope (SEM) studies. The epithelial tissue was carefully dissected and separated from the palate musculature. Samples of the palate tissue from each half were sectioned, frozen in liquid nitrogen and stored at -80°C for gelatinase zymography studies.

### Zymography

Samples of tissue, as well as mucus, were taken out of the freezer and ground to a powder in a mortar and pestal. Liquid nitrogen was added to keep the samples frozen. Homogenizing buffer (KCl 100 mM, ZnCl_2 _0.5 mM, EDTA 10 mM, Tris-HCl 1 M, pH 6.8) was added to the ground samples (approximately 500 ul buffer per 10 mg tissue sample, 200 buffer per 10 mg mucus sample) that were sonicated for 30 seconds on ice and then centrifuged at 8000 rpm for 5 minutes at 4°C. The supernatant was collected for protein assay (BCA protein assay kit, Pierce). Normalizing the protein content as 5 μg, different amount of samples were loaded into the 7.5% separating zymography gel (30% acrylamide/ 0.8% bisacrylamide 3.75 ml, 4 × Tris-Cl/ SDS, pH 8.8 3.75 ml, H_2_O 6 ml, 2% gelatin A 1.5 ml, 10% ammonium persulfate 0.1 ml, TEMED 0.01 ml, for 4 gels) and were run at 100 volts for 20 minutes, 150 volts for 40 minutes. After electrophoresis, the gel was washed 3 times (20 minutes per a time) in 2.5% Triton X-100 at room temperature followed by incubation for 96 hours in zymography development buffer (0.15 M NaCl, 5 mM CaCl_2_, 0.05% Azide NaN_3_, 50 mM Tris-HCl pH 7.6). The gel was then stained for 2 hours with stain solution (Coomassie brilliant blue R-250 1 g/L, methanol: acetic acid: H_2_O= 2.5: 1: 6.5) followed by de-staining (ethanol: acetic acid: H_2_O= 1: 2: 22) overnight. The expression of the gelatinases was shown on the gel as clear bands against the dark background stained with Coomassie blue. The bands were compared with the gelatinase standards for MMP 2 and 9 running in the first lane of each gel. The density of the gels was measured in the Bio-Rad scanning densitometer.

### Scanning electron microscopy

Samples of mucus and tissue were placed in 2.5 % glutaraldehyde solution immediately after collection and stored at 4°C until processing. The samples were post-fixed in 1 % osmium tetraoxide in Milonig's buffer at room temperature for one hour. They were then washed in a series of ethanol (50 – 100 %), ten minutes at each step, followed by two additional periods of absolute ethanol (10 minutes each). The samples were further dehydrated by critical point drying at 31°C for 5 – 10 minutes, then mounted on a specimen holder for SEM and dried overnight in vacuum desiccators. In the final stage of preparation for viewing, the samples were sputter coated with gold (Edwards, model S150B Sputter Coater). Samples were viewed using SEM (Hitachi S-2500). Images were scanned directly to a computer and stored as image files for subsequent viewing and analysis.

### Statistical analysis

Data are expressed as mean ± standard deviation unless otherwise stated. A paired Student-T test was used for simple comparison. The level of significance was set at 5 %.

## Results

The modified fresh frog palate exposure model was relatively easy to prepare and practical to handle. On gross examination, the surface of the palate exposed to side stream tobacco smoke did not show any macroscopic change in appearance after cigarette smoke exposure (CSE) compared to the control halves maintained in the normal chamber.

**Figure 3:**
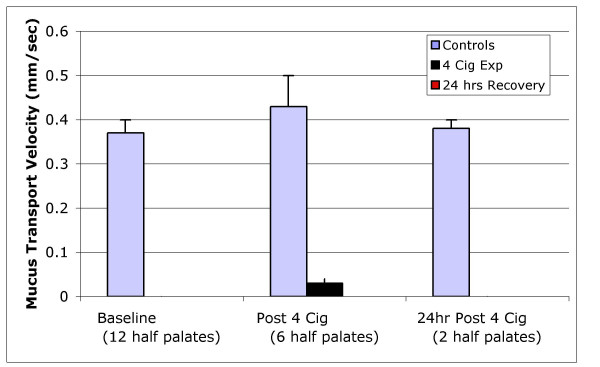
Three MTV controls are shown in the graph, no differences were observed among them. After immediate exposure to four cigarettes, mucus transport on the palates was drastically reduced. One of the four cigarette-exposed palate and its respective half palate control were maintained overnight in a refrigerator, and it was not possible to measure a transport time the next day in the exposed palate.

One half of the palate was used as a control and the other half was exposed to cigarette smoke. Baseline mucus transport velocity(**MTV**) was measured in both half palates prior to exposure of one half palate to cigarette smoke, and were identical (19.5 ± .03 mm/sec). Two additional MTV determinations were carried out in the control half after one and four cigarettes and after 24 h period of recovery during which time the palate was kept at 4°C. A paired T-test showed no statistical difference in mucus transport velocity among the control measurements during the entire experiment (Figure 3).

A paired T-test of MTV on the control half compared to the exposed half showed that mucus transport velocity in the exposed half palate was reduced (p < 0.03) immediately after exposure to the side stream smoke of one cigarette compared with the non-exposed half palate. Further exposure to the side stream smoke of three more cigarettes (4 in total) significantly reduced MTV (p < 0.001), with no signs of recovery after 24 hours.

We obtained a sample of mucus from the half palate exposed to four cigarettes and used it on the control half palate to measure MTV. Clearance was within the normal range. Immediately after, the same sample of mucus was tested on the exposed half palate and mucus clearance was again seen to be very slow. Mucus transport had basically ceased in some areas of the tobacco-smoke exposed palate.

Scanning electron microscopy (SEM) studies to assess the integrity of the epithelium after exposure to side stream smoke of one cigarette showed areas where the layer of cilia looked disordered. However, we did not see loss of cilia or exfoliation of ciliated epithelial cells after careful examination under the SEM in lower and high power of the entire surface of the sample after this level of exposure.

**Figure 4:**
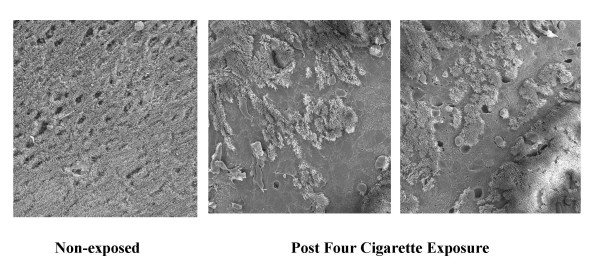
Magnification is ×400. On the left, the surface of the normal non-exposed palate is shown with a continuous ciliary layer, punctuated with secretory gland openings. The middle and right micrographs show the surface of palates exposed to the smoke of four cigarettes.

In Figure 4, SEM images from palates exposed to the smoke of four cigarettes showed greater epithelial tissue disruption (panels 2 and 3) compared to a control palate (panel 1). Large areas of deciliated cells were observed, as well as exfoliation of intact ciliated cells. Examples of exfoliated cells found on the surface of the epithelium in the representative SEMs are indicated with black arrows. Morphometric analysis of the area of cilia loss from 3 paired palates exposed to four cigarettes, evaluating 12 different areas randomly selected in each palate, showed cilia loss of 51 ± 14 % compared to < 2 % on control palates.

**Figure 5:**
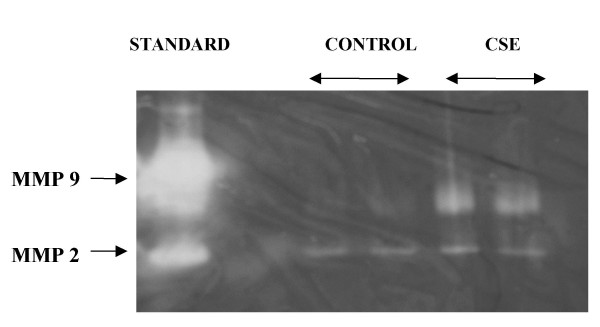
A representative gelatinase zymogram on mucus collected from control and cigarette-exposed palates. In the left lane, standards for MMP 2 and 9 are shown. In the next two lanes MMP activity is shown in control mucus (two samples). The next two lanes show MMP activity in two aliquots of the mucus collected from palates exposed to the smoke of four cigarettes.

Gelatinase zymography showed increased activity of MMP-9 in mucus collected from the palates exposed to tobacco smoke of four cigarettes compared to mucus from control palates as shown in Figure 5. MMP2 activity was not different in mucus samples obtained from palates exposed, or not to cigarette smoke, but these results are inconclusive.

## Discussion

Major findings in this study include: a) our amphibian mucociliary clearance model appears to be appropriate to study the early effects of tobacco smoke exposure; b) the clearance of mucus is significantly reduced after exposure to side stream tobacco smoke associated to dose response; c) ciliated cells are anatomically and physiologically acutely affected therefore drastically impairing mucus clearance. Acute irritation and inflammation of cilia due to exposure to tobacco smoke could explain this alteration; d) mucus seems to not be physiologically affected at this stage; and e) tobacco smoke triggers an increased activity of matrix metalloproteinases (in particular MMP9) associated with disruption of the extracellular matrix, most likely affecting cellular attachments to the basal membrane generating a substantial exfoliation of the ciliated cells.

These findings might assist us in enhancing the knowledge of the changes in the mucociliary apparatus that occur early after exposure to environmental tobacco products, with the goal to understand how these changes relate to the development of chronic airway pathologies in humans.

The frog palate has been used for several decades as a model to assess mucociliary clearance [13-17]. Different species of frogs have also been used, as well as a variety of study designs. Researchers had to deal with several sources of variability, making it difficult to standardize a widely acceptable model. There are disadvantages in our model such that it pertains to a non-mammalian species, in addition that the epithelial tissue is non-respiratory. However as a model it has advantages over some mammalian models like rodents in that the ciliated epithelium has a well developed mucus blanket that work in coordination with cilia similar to the human situation.

This exposure model is an isolated system, theoretically free of interferences from other agents or systemic physiological responses resulting from other internal or external influences. In this injury model we randomly exposed either half palate to side stream tobacco smoke and used the opposite half palate as the control (internal control). Since there was no statistical difference in mucus transport between them, previously established in different sets of pairs of palates, one side was arbitrarily selected for control and the opposite half for exposure to tobacco smoke. This study follows an approach utilized previously by Zayas *et al *[7] that compared samples of mucus obtained from both mainstem bronchi in smokers and in nonsmokers.

In assessing the mucus transport rate in both half palates before any experimental procedure and to insure that they functioned identically, we established baseline data for our experiment. This helped to reduce error variability due to sex, weight, age, as well as control for any seasonal effect on the mucus transport rate in the frog palates as demonstrated by Rubin *et al *[18].

Since the palate is excised from the frog we may assume that any observed effect or response is a local and direct effect of exposure to side stream tobacco smoke, which is an advantage of our model. From the data obtained, we would conclude that our frog palate exposure model is suitable to study the acute effects of second hand tobacco smoke.

We may also conclude that clearability of mucus seems not to be altered at this stage, at this level of exposure and in this particular exposure preparation or design. Therefore, at this stage mucus transport seems not to be functionally affected for being cleared in a normal fashion by non-exposed cilia. However, after more prolonged tobacco exposure there will be modifications in mucus properties, as seen and measured in smoking dogs where after two months of tobacco exposure, the galactose content and the viscoelastic properties of mucus were observed to present alterations. After further exposure the viscosity and elasticity properties of mucus were reversed to quasi-normal levels, but galactose content did not [12]. Hence the model makes it very useful for differentiation of cilia-related effects versus mucus-related effects on mucociliary clearance after acute tobacco exposure.

Mucus clearance is a function of cilia beat frequency and mucus viscoelastic properties. A change in mucus clearance may be due to one or the other component or both. In future studies, incorporating high speed digital imaging of the palate surface to determine cilia beat frequency will allow us to further differentiate between cilia related effects versus mucus related effects.

Exposure to tobacco smoke with all its noxious agents and components will possibly allow us to separate and study in detail, the timing and appearance of different phases of the organism response. Our exposure model may allow us to individualize and study the inflammation phase resulting from the tobacco exposure. We can then focus on the injury phase occurring in the mucociliary system. Later we will explore mechanisms involved in the healing process. The remodeling of the ciliated epithelium following acute injury will be an important component of our studies and particularly how the remodeling is mediated. The time frame of the injury and recovery phases needs to be determined.

Cilia do not work alone, but in association with other cilia to produce metachronal waves. Several metachronal waves may contribute to the propulsion of the mucus layer to flow over irregularities or non-ciliated areas. Our data indicates that acute tobacco exposure may have an initial and early irritation effect, possibly mediated by an unknown mechanism on the exposed palate that leads to inhibition of ciliary beat frequency or discoordination of metachronal waves. Such factors adversely affect clearance by imposing stasis or local eddies resulting in erratic clearance that may be the reason why scanning electron micrographs of palates after one cigarette showed cilia somewhat disordered, but not visibly disrupted.

We have shown that tobacco smoke exposure interferes with mucociliary clearance. Sustained exposure may lead to loss of ciliated epithelium associated with activation of matrix metalloproteinases. Significant loss of cilia or ciliated epithelial cells results in disruption or even cessation of mucociliary clearance. Matrix metalloproteinases may be implicated in this injury through disruption of epithelial cell-to-cell or cell-to-basement membrane connections. Further clarification of the mechanisms involved will be undertaken in subsequent studies.

Our exposure model can assess physiological, ultrastructural and molecular parameters in response to the initial deleterious effects of acute exposure to side stream tobacco smoke in an epithelial model homologous to the human airways. In these preliminary studies we did not attempt to mimic "human smoking conditions". However, our results show that MTV is affected even after exposure to one cigarette. Although concentration of smoke used in the present study is higher than likely encountered in typical environmental tobacco exposures, they are within an order of magnitude of those computed for exposure in poorly ventilated cars or homes. Future studies will try to replicate real conditions faced by non-smokers exposed to environmental tobacco smoke and to characterize the mediation and effectors of this acute injury.
